# Targeting Drug Resistance in Cancer: Dimethoxycurcumin as a Functional Antioxidant Targeting ABCC3

**DOI:** 10.3390/antiox14050599

**Published:** 2025-05-16

**Authors:** Jochem Nelen, Valeria Naponelli, José Manuel Villalgordo-Soto, Marco Falasca, Horacio Pérez-Sánchez

**Affiliations:** 1 Structural Bioinformatics and High Performance Computing Research Group (BIO-HPC), HiTech Innovation Hub, UCAM Universidad Católica de Murcia, 30107 Murcia, Spain; jnelen@ucam.edu; 2 Health Sciences PhD Program, Universidad Católica de Murcia UCAM, Campus de los Jerónimos n°135, Guadalupe, 30107 Murcia, Spain; 3 University of Parma, Department of Medicine and Surgery, Via Volturno 39, 43125 Parma, Italy; valeria.naponelli@unipr.it; 4 Eurofins-Villapharma Research, 30320 Murcia, Spain; josemanuel.villalgordo@discovery.eurofinseu.com

**Keywords:** cancer drug resistance, virtual screening, curcumin derivatives, molecular fingerprints, pancreatic cancer, ABC transporters, antioxidants, natural products

## Abstract

The development of new anticancer therapies remains challenging due to tumor heterogeneity and the frequent emergence of multidrug resistance (MDR). Natural products have garnered increasing attention as alternative or complementary therapeutic agents due to their bioactivity and reduced toxicity. Polyphenols, particularly curcumin and its derivatives, have shown promise in modulating signaling pathways, enhancing chemosensitivity, and overcoming drug resistance. The anticancer potential of dimethoxycurcumin, a chemically modified curcumin derivative identified through consensus fingerprint similarity screening, was investigated for its potential to inhibit ABCC3 (MRP3)—a member of the ATP-binding cassette (ABC) transporter family implicated in drug efflux, tumor cell survival, and resistance. In vitro experiments demonstrated that dimethoxycurcumin significantly reduced cancer cell viability and colony formation, indicating a strong inhibitory effect on ABCC3 function. These results suggest that dimethoxycurcumin may sensitize cancer cells to chemotherapy by targeting resistance pathways. The data presented contribute to the growing body of evidence suggesting that bioactive plant-derived compounds, including chemically modified derivatives, may hold therapeutic potential in oncology by modulating multidrug resistance pathways. Targeting ABC transporters with natural compound derivatives could offer a promising strategy for developing more effective and less toxic anticancer therapies.

## 1. Introduction

Advancing anticancer therapies is a significant challenge due to the intricate nature of tumor biology and the persistent issue of drug resistance [1]. While conventional small-molecule drugs have been highly effective in cancer treatment, their long-term use can be limited by toxicity, off-target effects, and the emergence of multidrug resistance (MDR). MDR arises through various mechanisms, including enhanced drug efflux, metabolic adaptation, and alterations in drug targets, ultimately reducing therapeutic efficacy [2]. This issue is especially critical in pancreatic ductal adenocarcinoma (PDAC), an aggressive malignancy with one of the highest cancer-related mortality rates. Its poor prognosis stems from multiple factors, including the rapid emergence of drug resistance, which significantly limits treatment effectiveness [3].

ABC transporters play a crucial role in cancer resistance by actively exporting drugs from cells, reducing intracellular concentrations, and promoting chemoresistance [4]. These membrane transporters are involved in various mechanisms of drug resistance, affecting the efficacy of many chemotherapeutic agents, including cisplatin and taxanes [5]. One notable member of this family is ATP-binding cassette subfamily C member 3 (ABCC3), also known as multidrug resistance-associated protein 3 (MRP3). ABCC3 plays a key role in drug disposition and has been implicated in cancer cell survival and proliferation, particularly in pancreatic cancer [6]. In addition to its role in drug resistance, it has been found that ABC transporters actively contribute to cancer progression by extruding bioactive molecules that can influence the tumor microenvironment [7,8,9,10]. Their role in modulating drug response and promoting cancer progression makes them a relevant target for potential therapeutic interventions. Strategies to counteract ABC transporter-mediated resistance include the development of small-molecule inhibitors, combination therapies to enhance drug retention, and natural compound-based approaches that modulate transporter activity [11]. Targeting these mechanisms could improve chemotherapy efficacy and help overcome treatment resistance in cancers such as PDAC, where drug efflux plays a significant role in therapeutic failure. Notably, ABCC3 inhibitors have the potential to not only enhance drug retention but also impede tumor progression by blocking the extrusion of bioactive molecules that contribute to the tumor microenvironment and cancer cell survival [10].

The challenges of drug resistance in cancer have led to growing interest in alternative strategies, such as natural product-based compounds, which may complement existing treatments by targeting resistance pathways while offering potentially improved safety profiles [12]. Plant-derived bioactive compounds, particularly polyphenols such as flavonoids [13], hold promise in oncology due to their antioxidative effects, regulation of signaling pathways, and potential to enhance chemosensitivity [14]. Antioxidants play a crucial role in mitigating oxidative stress, a factor implicated in cancer progression and resistance to therapy [15]. Their ability to modulate the tumor microenvironment, influence immune responses, and synergize with conventional treatments could make them valuable in cancer management. Several plant-derived compounds have already demonstrated clinical success, underscoring the importance of further exploration in this area. For instance, paclitaxel, a taxane originally extracted from *Taxus brevifolia*, is widely used to treat various malignancies, including breast, ovarian, and lung cancers [16]. Similarly, vinblastine and vincristine, alkaloids derived from *Catharanthus roseus*, are essential components of chemotherapy regimens for hematologic malignancies and solid tumors [17]. Additionally, camptothecin, isolated from *Camptotheca acuminata*, has led to the development of topoisomerase inhibitors such as irinotecan and topotecan, which are used in colorectal, ovarian, and small-cell lung cancers [18]. These examples highlight the immense potential of plant-derived compounds in oncology and reinforce the need to investigate novel bioactive molecules with improved efficacy and safety profiles. Beyond these already well-established plant-derived chemotherapeutics, other natural compounds continue to attract attention for their potential anticancer properties.

Taken together, these findings highlight the urgent need for novel therapeutic strategies capable of overcoming multidrug resistance in PDAC by targeting key molecular mechanisms such as ABC transporter activity. In particular, ABCC3 (MRP3) represents a compelling target due to its established role in drug efflux and tumor progression. To explore potential inhibitors, a ligand-based virtual screening campaign was carried out using a structurally diverse compound library, including both synthetic molecules and natural product derivatives. This approach aimed to identify small-molecule modulators of ABCC3 that may enhance chemosensitivity and contribute to improved therapeutic outcomes in pancreatic cancer.

## 2. Materials and Methods

### 2.1. Virtual Screening Using Consensus Fingerprint Similarity

The diverse and proprietary in-house Eurofins-VillaPharma library was processed to generate three types of molecular fingerprints: Avalon [19], Circular (Extended Connectivity Fingerprint with a diameter of 6, ECFP6) [20], and PubChem Substructure fingerprints [21], using the PyFingerprint package (version 3.0) [22]. This package leverages the RDKit [23] for Avalon fingerprint generation, and the Chemistry Development Kit (CDK) [24] for PubChem and Circular (ECFP6) fingerprints. As part of the fingerprint conversion process, the input molecules were sanitized and pre-processed, including steps such as charge normalization, salt removal, and tautomer standardization, to ensure consistent and reliable representations. The resulting fingerprints were encoded as binary vectors, which enabled efficient and rapid similarity calculations for downstream analyses.

Initially, a custom script was developed to compare the query structure against the library. This script converted the input query on-the-fly into Avalon, PubChem, and Circular (ECFP6) fingerprints and subsequently compared them to the corresponding fingerprints in the Eurofins-VillaPharma library using the Euclidean distance metric. To ensure comparability across fingerprints, the distances were normalized using z-score normalization, where each value was scaled based on the mean and standard deviation of its distribution, resulting in a standardized score for each fingerprint type.

The final consensus score for each compound was obtained by averaging these normalized scores across all three fingerprint types. Compounds were then ranked based on their consensus scores, with higher-ranking compounds selected for further evaluation. This consensus approach attempts to reduce fingerprint-specific biases and provide a more robust prediction of potential hits, while also increasing the chemical diversity among the top-ranked compounds.

To facilitate reproducibility and improve usability, this initial screening script was further developed into ConFiLiS v1.0 (Consensus Fingerprints for Ligand-based Screening), an open-source tool that refines the original methodology with improved preprocessing and performance, while maintaining the core principles of the original approach. The tool, along with detailed documentation and usage instructions, is publicly available on GitHub: https://github.com/Jnelen/ConFiLiS (accessed on 27 March 2025).

### 2.2. Hit Comparison Against BindingDB

To assess the structural novelty of the identified hits, all known ABCC3-active compounds were retrieved from BindingDB [25] and converted into Morgan fingerprints (radius 2, 2048 bits) using RDKit. The Morgan fingerprinting method, which is based on extended-connectivity circular fingerprints (ECFP), was chosen for its ability to effectively capture molecular substructures and functional groups that contribute to biological activity, making it particularly suitable for structural similarity analysis in drug discovery.

To quantify structural similarity, Tanimoto similarity coefficients were computed between each hit and all known ABCC3-active compounds present in the BindingDB dataset. The Tanimoto coefficient, a widely used metric in cheminformatics, measures the degree of overlap between two molecular fingerprint representations, yielding a numerical value between 0 (no similarity) and 1 (identical structures). This analysis enabled the identification of the closest known ABCC3-associated compound in BindingDB for each hit based on structural similarity. This step was critical in determining whether the identified hits represented novel chemical scaffolds or bore a close resemblance to previously reported inhibitors. Following the computational similarity analysis, a manual review was performed to evaluate the similarity of each hit to the closest known ABCC3-associated compounds. This curation step was essential for prioritizing candidates with novel chemical scaffolds, thereby minimizing the risk of rediscovering previously known inhibitors while maximizing the potential for identifying structurally distinct ABCC3-targeting compounds.

In addition to the structural similarity assessment, in silico ADME property predictions were carried out to evaluate the drug-likeness and pharmacokinetic profiles of the selected compounds. SMILES representations of the hits were converted to SDF format using RDKit and imported into Schrödinger’s Maestro (version 2024-3) [26]. Explicit hydrogen atoms were added, and 3D structures were generated using the Maestro interface. The QikProp module [27] was subsequently used to calculate key physicochemical and pharmacokinetic parameters, including predicted logP, aqueous solubility, and human oral absorption.

Finally, molecular docking was performed to investigate the potential binding modes of the prioritized hit compounds. Specifically, blind docking was conducted using AutoDock Vina [28] through MetaScreener [29], targeting all α-carbon atoms across the protein structure to identify potential binding sites without prior knowledge of specific active regions [30]. The resulting docking poses were clustered based on spatial proximity, and the most populated clusters were examined to identify key binding hotspots. From these, the top-scoring pose from the cluster within the main channel was selected for protein–ligand interaction analysis. To characterize the predicted binding interactions, the selected protein–ligand complex was processed using Protein–Ligand Interaction Profiler (PLIP) [31]. Molecular docking poses and PLIP interaction diagrams were rendered using PyMOL (version 2.6.2) [32], to produce high-resolution figures for visualization.

### 2.3. Viability Assays

HPAF-II, BxPC-3, and CFPAC-1 cells were seeded in 96-well plates at a density of 5000 cells per well and allowed to adhere for 24 h under standard culture conditions (37 °C, 5% CO_2_). Cells were then treated with the indicated compounds at varying concentrations for an additional 72 h to assess dose-dependent effects on viability.

Following the treatment period, cell viability was evaluated by measuring the metabolic activity of live cells. The cells were first fixed with 3% paraformaldehyde for 10 min at room temperature to preserve morphology and prevent detachment. They were then stained with 0.5% crystal violet for 15 min to label adherent cells. Excess dye was removed by gently washing the wells with deionized water, and the plates were left to air-dry completely.

To quantify cell viability, the bound crystal violet dye was solubilized using 0.1 M sodium citrate, and absorbance was measured at 590 nm using a microplate reader (EnSpire, PerkinElmer, Waltham, MA, USA). The optical density (OD) values obtained reflected the relative number of viable, adherent cells, providing a quantitative measure of treatment efficacy. All experiments were performed in triplicate to ensure reproducibility, and background absorbance was subtracted using wells containing only the staining solution. Graphics and IC_50_ calculations were carried out using GraphPad Prism v10.0.

### 2.4. Clonogenic Assays

BxPC-3 cells were seeded in 6-well plates at a density of 500 cells per well and incubated in a complete medium at 37 °C in a humidified atmosphere containing 5% CO_2_ for 12 days to allow colony formation. The medium was refreshed every three days to maintain optimal growth conditions. For treatment experiments, the medium was supplemented with the indicated concentrations of DMSO or test compounds, ensuring continuous drug exposure throughout the incubation period. After 12 days, colony formation was assessed by first fixing the cells with 4% paraformaldehyde for 20 min at room temperature to preserve the cellular structure and prevent detachment. The colonies were then stained with 0.5% crystal violet solution for 30 min to enhance visualization. Excess dye was removed by gently rinsing the wells multiple times with deionized water, and the plates were air-dried.

Colony formation was visualized using a bright-field microscope, and images were captured for documentation. Colonies were manually counted to quantify clonogenic potential. Alternatively, to achieve higher sensitivity and automated quantification, fixed colonies were stained with high-content screening (HCS) CellMask™ Deep Red (cat. n. H32721, Thermo Fisher Scientific, Eugene, OR, USA) and 4′,6-diamidino-2-phenylindole (DAPI, cat. n. D1306, Thermo Fisher Scientific, Eugene, OR, USA). Fluorescent images were acquired and analyzed using the IN-Cell Analyzer 2200 (GE Healthcare Life Sciences, Marlborough, MA, USA) to provide a more precise and reproducible assessment of colony formation.

All experiments were conducted in triplicate to ensure statistical reliability, and appropriate controls were included to account for background staining and autofluorescence.

### 2.5. Statistical Analysis

Results are presented as mean ± standard error of the mean (SEM). One-way ANOVA was performed to evaluate the significance of differences between treatment groups and the control group, followed by Dunnett’s post hoc test. Statistical analyses were conducted using GraphPad Prism version 10.0, and a *p*-value < 0.05 was considered statistically significant.

## 3. Results

### 3.1. Consensus Fingerprint-Based Virtual Screening for ABCC3 Inhibitor Discovery

Potential ABCC3 inhibitors were identified using a consensus fingerprint-based virtual screening approach using known ABCC3-active compounds from BindingDB as a reference. Compound BDBM50302828, with a reported IC_50_ of 1.1 µM, served as the starting query structure. At the time of selection, it was the most potent small-molecule drug for ABCC3 listed in BindingDB, making it the most suitable candidate.

To maximize chemical diversity and ensure broad structural coverage, three types of molecular fingerprints—Avalon, Circular (ECFP6), and PubChem substructure fingerprints—were generated for both the query compound and the screening library. These fingerprints were subsequently used to calculate Euclidean distances between the reference compound and each library entry.

A consensus score was derived by averaging the normalized distances across all three fingerprint types, ensuring that hits were not disproportionately influenced by any single fingerprinting method. This approach prioritizes compounds that exhibit a consistent degree of similarity across multiple fingerprinting techniques, thus improving the likelihood of identifying true ABCC3 inhibitors rather than false positives arising from fingerprint-specific artifacts.

For the final selection, a consensus score cutoff of −2.5 was applied, meaning that only compounds with the highest overall structural similarity to the reference compound across all fingerprint types were considered for further evaluation. Applying this threshold resulted in five top-ranked compounds, which were selected for experimental validation. This threshold was chosen to balance hit diversity with structural relevance to focus on candidates with high potential for ABCC3 inhibition while maintaining chemical diversity. Selected hits were subsequently subjected to further computational and experimental validation to assess their potential as novel ABCC3 inhibitors.

Table 1 summarizes the Euclidean distances for each fingerprint type, along with their normalized counterparts (Dist and Norm, respectively). The consensus score was computed as the average of these normalized distances and served as the basis for the final ranking of compounds. Only compounds with a consensus score below −2.5 were considered for further testing.

**Figure 1:**
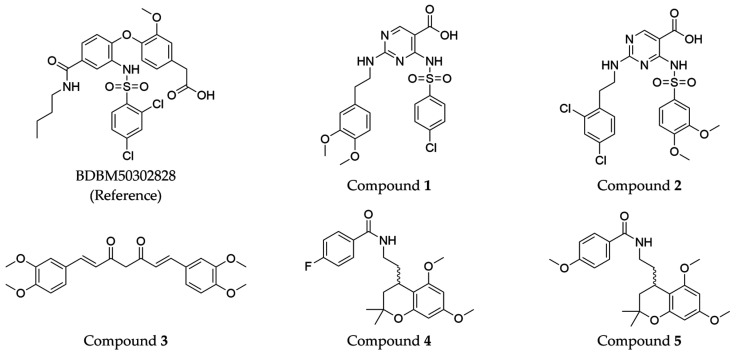
Chemical structures of predicted ABCC3 inhibitors. The reference compound, BDBM50302828, is presented alongside the five top-ranking compounds selected for testing. Compound **3** was identified as dimethoxycurcumin, a methylated natural derivative of curcumin, while Compounds **1**, **2**, **4**, and **5** are structurally diverse synthetic compounds without natural product origins. Compounds **4** and **5** were synthesized as racemic mixtures, as indicated by the wavy bond notation.

The compound structures, along with the BDBM50302828 reference compound, are shown in Figure 1. It is noteworthy that these five compounds represent three distinct scaffolds. Compounds **1** and **2** share structural features, including a sulfonamide moiety, which is also present in the reference compound. Additionally, Compounds **4** and **5** feature a 5,7-dimethoxy-2,2-dimethylchromane-based scaffold linked to a benzamide moiety.

This structural diversity suggests that different scaffolds capture distinct features from the query structure, potentially interacting with ABCC3 through varied binding modes. Notably, Compound **3**, identified as dimethoxycurcumin, exhibits a high degree of structural similarity to curcumin, differing only by the methylation of both hydroxyl groups into methoxy groups.

In summary, the screening approach yielded a set of structurally diverse hits, underscoring the potential of consensus fingerprint similarity techniques in virtual screening workflows. The observed chemical diversity among the top-ranked hits highlights the utility of integrating multiple fingerprint types to mitigate model-specific biases and expand the structural search space.

### 3.2. BindingDB Similarity Analysis

To gain a deeper understanding of the structural characteristics of the identified hits, a comparison was made against known ABCC3-active compounds from BindingDB. This analysis aimed to uncover potential structural relationships between the predicted hits and previously reported ABCC3 inhibitors. In total, 1849 ABCC3-related entries were retrieved and used as a reference set. Each hit was compared to this reference set by calculating Tanimoto similarity scores using Morgan (ECFP4) fingerprints, and the closest known compound was identified for each hit.

The results of this similarity analysis are compiled into Table 2, which lists each predicted hit, its closest known ABCC3 compound, and the corresponding Tanimoto similarity score calculated using the Morgan (ECFP4) fingerprint. A subsequent manual assessment was performed to evaluate the distinctiveness of each hit, ensuring that structurally novel candidates were prioritized for further testing while minimizing the likelihood of rediscovering known inhibitors.

None of the predicted hits exactly matched any known ABCC3 inhibitors deposited in BindingDB. Compounds **1** and **2** were both structurally closest to the same compound, BDBM50012957, with Tanimoto similarity scores of 0.3810 and 0.3222, respectively. This structural similarity is likely due to the presence of a common sulfonamide moiety. Compound **3**, also known as dimethoxycurcumin, exhibited the highest similarity to any of the known ABCC3-active compounds, with a Tanimoto similarity of 0.5556. Although this similarity is relatively high, the structure was deemed sufficiently distinct to warrant testing, given the presence of amide and benzoic acid moieties in the BindingDB compound, which are not found in the predicted hit compound. Furthermore, the natural product-like characteristics of this compound contributed to its selection for further investigation. Finally, Compounds **4** and **5** were found to be the most distinct from any of the known compounds, with Tanimoto similarities of 0.2637 and 0.2703, respectively. Interestingly, despite their high structural similarity—differing only by the presence of either a fluoro or methoxy group on the para position of the terminal benzene ring—their closest known compounds were different.

To further evaluate the drug-likeness and pharmacokinetic profiles of the selected hits, in silico ADME predictions were conducted using Schrödinger’s QikProp module [27]. Key physicochemical and pharmacokinetic parameters, such as predicted logP, aqueous solubility, and human oral absorption, are presented in Table 3. These results provide insights into the predicted drug-likeness and pharmacokinetic behavior of the selected compounds.

**Figure 2:**
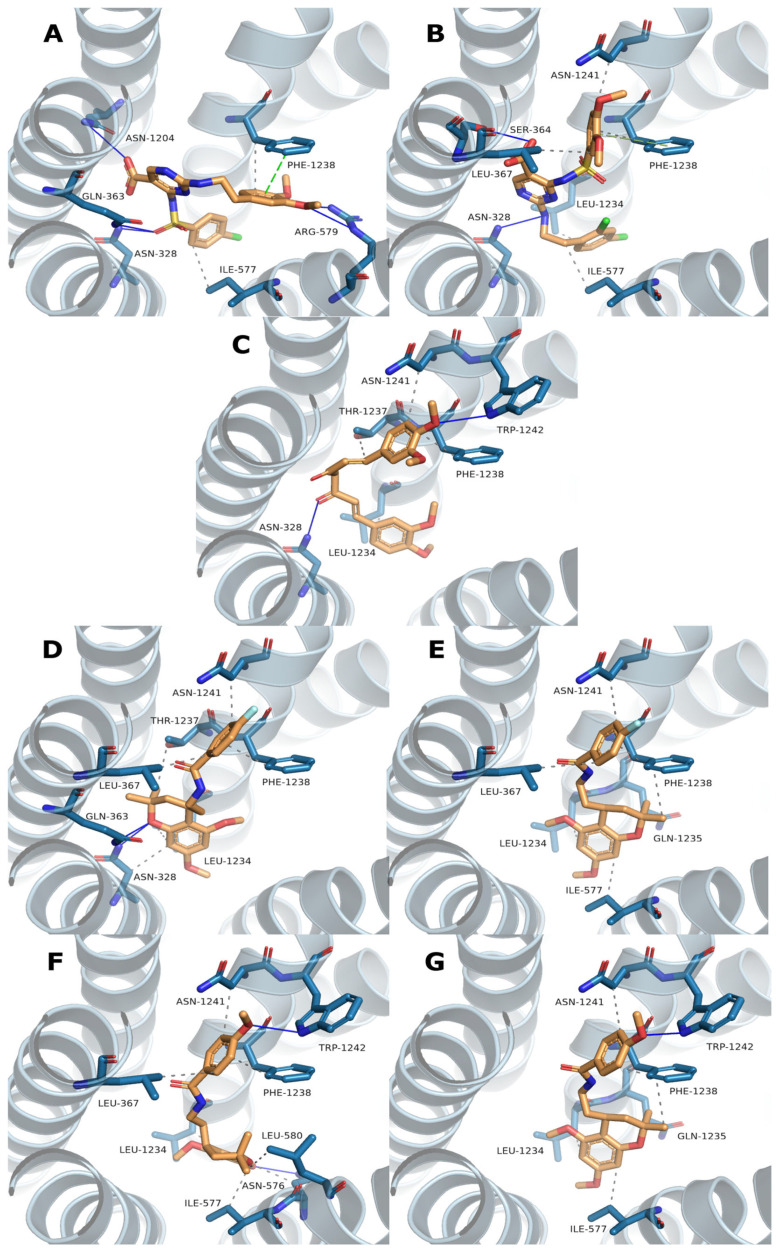
Predicted binding poses and interaction profiles of selected ABCC3 hit compounds. AutoDock Vina blind docking identified top-ranked poses in the ABCC3 transporter’s main channel. Key non-covalent interactions—hydrogen bonds (solid lines), hydrophobic contacts (gray dashed), and π–π stacking (green dashed)—were identified using PLIP. Panels (**A**–**C**) show Compounds **1**–**3**; (**D**,**E**) and (**F**,**G**) depict the R and S enantiomers of Compounds **4** and **5**, respectively.

To further characterize the selected hit compounds beyond structural similarity and ADME properties, molecular docking was performed to predict their potential binding modes within the ABCC3 transporter. Blind docking using AutoDock Vina enabled an unbiased search for potential binding sites across the protein surface. Among the docking results, the highest-scoring pose located within the main channel was selected as the most probable binding mode. To gain structural insight, interaction profiles were generated using the Protein–Ligand Interaction Profiler (PLIP), which can identify and visualize hydrogen bonds, hydrophobic contacts, and other relevant non-covalent interactions. The resulting binding poses and interaction maps are presented in Figure 2, providing a visual summary of the predicted docking poses and interactions for each compound. Table 4 provides a summary of the interacting residues along with the corresponding Vina scores for all docking poses.

### 3.3. Determine Activity Using Cellular Assays

**Figure 3:**
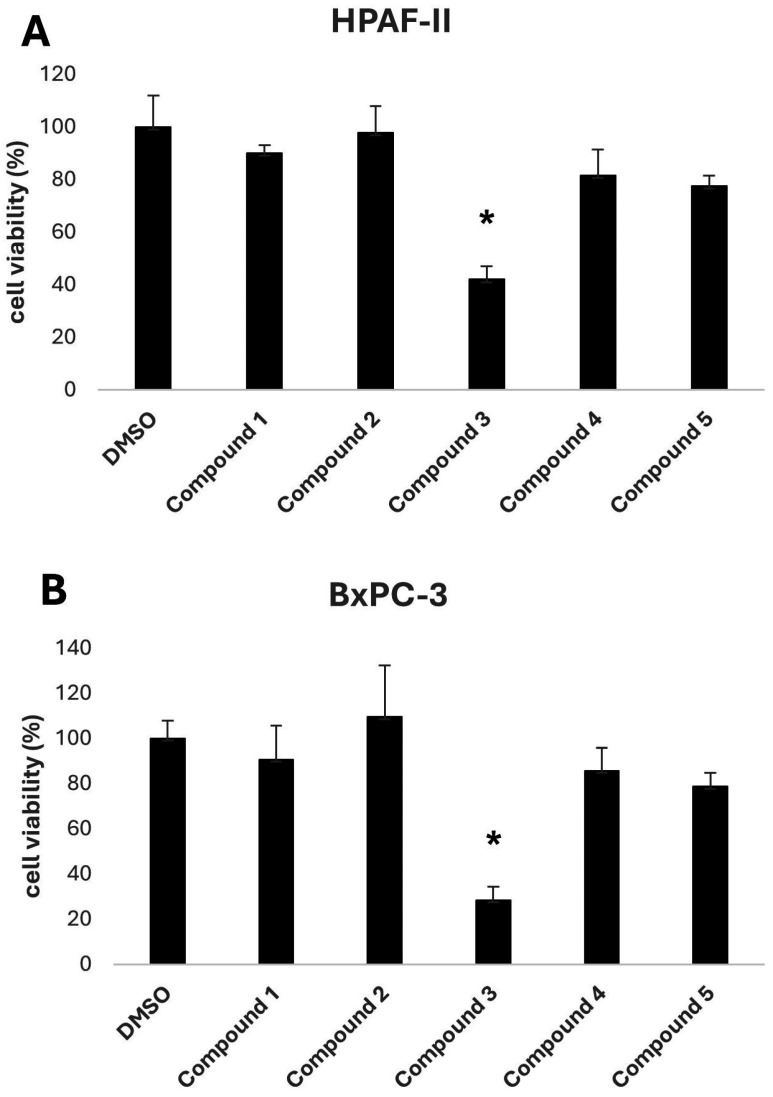
Cell viability of HPAF-II (**A**) and BxPC-3 (**B**) cells after 72 h treatment with 10 μM of Compounds **1**–**5** or DMSO (vehicle control). Viability was assessed by crystal violet staining and is expressed as a percentage. Data are presented as mean ± SEM from three independent experiments. Statistical analysis was performed using repeated measures of one-way ANOVA, followed by Dunnett’s post hoc test to compare treatments to the control. * *p* < 0.05 was considered statistically significant.

The biological activity of the five selected compounds was evaluated using a panel of human pancreatic cancer cell lines, including HPAF-II, BxPC-3, and CFPAC-1. To assess their impact on cell growth, an initial screening was performed by treating the cells with a fixed concentration of 10 μM for HPAF-II and BxPC-3, as shown in Figure 3. Among the tested compounds, Compound **3**, a methylated derivative of curcumin, exhibited the highest activity.

**Figure 4:**
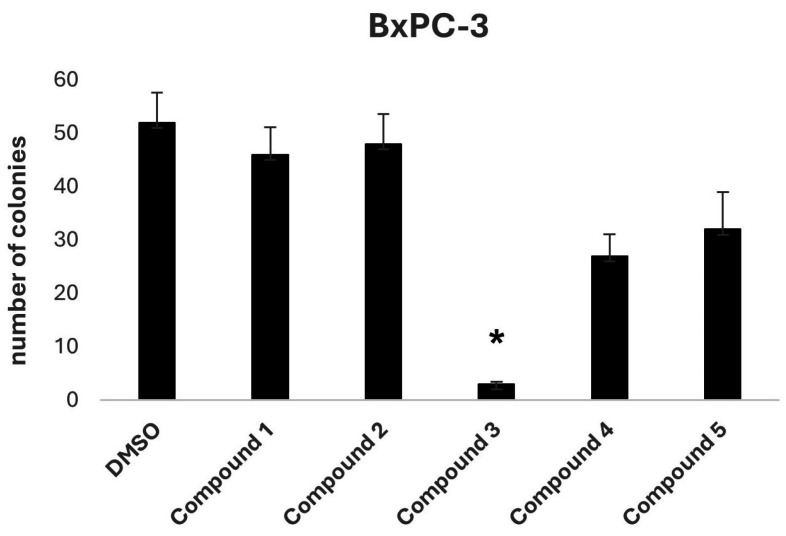
Effects of tested compounds (10 μM) on BxPC-3 cell colony formation. Cells were treated with 10 μM of each compound (Compounds **1**–**5**) or DMSO (vehicle control) and incubated for 12 days. Data are presented as mean ± SEM of three independent experiments. Statistical analysis was performed using repeated measures of one-way ANOVA, followed by Dunnett’s post hoc test to compare treatments to the control. * *p* < 0.05 was considered statistically significant.

To further investigate the potential anti-tumorigenic properties of the selected compounds, a two-dimensional colony formation assay was conducted using the BxPC-3 cell line (Figure 4). At a concentration of 10 μM, Compound **3** again stood out as the most effective candidate, completely inhibiting colony formation, indicating its potential for disrupting cancer cell proliferation.

**Figure 5:**
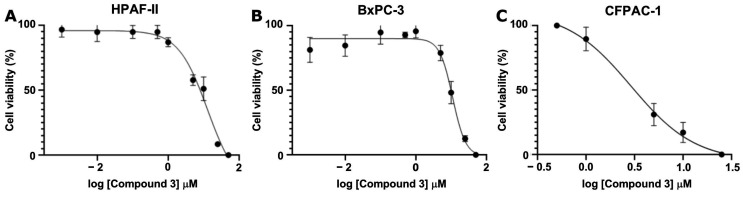
Dose-Response curve of Compound **3** for IC_50_ determination in (**A**) HPAF-II (0.001–50 µM), (**B**) BxPC-3 (0.001–50 µM), and (**C**) CFPAC-1 (0.1–25 µM) cell lines. Data are presented as mean ± SEM of the three independent experiments.

To further characterize its potency, Compound **3** was tested across a range of concentrations (1 nM to 50 μM) to determine the IC_50_ value using a crystal violet viability assay for three different cancer cell lines: HPAF-II, BxPC-3, and CFPAC-1. Compound **3** demonstrated substantial growth inhibition, yielding IC_50_ values of 11.03 μM, 12.90 μM, and 2.91 μM for HPAF-II, BxPC-3, and CFPAC-1, respectively (Figure 5).

## 4. Discussion

Multidrug resistance (MDR) remains a central obstacle in effective chemotherapy, particularly in aggressive solid tumors such as pancreatic ductal adenocarcinoma (PDAC). Central to this resistance mechanism are ATP-binding cassette (ABC) transporters, with ABCC3 (MRP3) emerging as a critical mediator of chemotherapeutic drug efflux and tumor progression. Identifying novel and potent modulators of ABCC3 is, therefore, crucial in advancing therapeutic strategies to overcome resistance and improve patient outcomes.

ConFiLiS, a virtual screening tool employing consensus fingerprint similarity scoring, was used to identify structurally diverse inhibitors of ABCC3 from the proprietary Eurofins-VillaPharma compound library. By integrating multiple molecular fingerprint types—Avalon, ECFP6, and PubChem substructure—this method mitigated fingerprint-specific biases and facilitated the discovery of structurally varied candidates with potential pharmacological activity. The resulting compounds fall into three distinct chemotypes: sulfonamide-containing molecules (Compounds **1** and **2**), dimethoxy-2,2-dimethylchromane-based scaffolds linked to a benzamide moiety (Compounds **4** and **5**), and a methylated curcumin derivative, dimethoxycurcumin (Compound **3**).

Structural similarity analysis against known ABCC3 ligands in BindingDB reinforced the novelty of the predicted compounds. Dimethoxycurcumin demonstrated a relatively high structural similarity to a known ABCC3-active compound (Tanimoto coefficient 0.5556), yet retained distinct pharmacophoric differences, particularly the absence of amide and benzoic acid functionalities, which supported its further investigation. The observed structural differences, despite sharing common molecular features with known inhibitors, underscore the chemical novelty achievable through unbiased ligand-based screening, emphasizing subtle yet potentially impactful modifications that could affect biological activity or potency.

To further guide early evaluation, key physicochemical and pharmacokinetic properties were predicted using QikProp (Table 3). All selected candidates exhibited general favorable drug-like characteristics, including high predicted human oral absorption, acceptable solubility profiles, and minimal violations of Lipinski’s and Jorgensen’s rules. These in silico predictions served as a screening filter to identify any major outliers that might warrant caution before biological testing. However, all compounds fell within acceptable ranges and were, therefore, included in downstream assays.

Among the tested ligands, Compound **3** (dimethoxycurcumin) displayed the most favorable overall profile, combining high solubility, strong permeability, and zero rule-of-five and rule-of-three violations. In contrast, compounds **1** and **2** showed notably low Caco-2 cell permeability and reduced predicted oral absorption (71% and 57%, respectively), which may indicate potential absorption limitations in vivo. Compound **5** and especially Compound **4** exhibited relatively low aqueous solubility, with values predicted to fall below the typical range measured for most drugs, which could suggest potential formulation challenges. However, predicted permeability and oral absorption were favorable, warranting inclusion in the experimental studies.

To further evaluate the binding mode of the selected compounds, molecular docking was performed using a blind docking approach with AutoDock Vina. Although in some cases the highest-scoring pose corresponded to an allosteric site, we deliberately selected poses within the central translocation channel of the ABCC3 transporter due to their greater biological relevance. Importantly, the difference in docking scores between the allosteric and channel-bound poses was minimal (<0.3 kcal/mol), supporting the validity of this selection. Within the channel, all compounds were placed in a similar binding region despite differences in chemical structure (Figure 2), suggesting a common site of interaction. Still, the predicted binding poses themselves were quite diverse in orientation and contact patterns, indicating flexibility in how different ligands may engage the site. Interestingly, the R and S configurations of Compounds **4** and **5** were predicted to adopt binding poses with a highly similar orientation of the benzamide moiety, while the 5,7-dimethoxy-2,2-dimethylchromane scaffold at the stereocenter displayed nearly flipped positions. Interaction profiling using PLIP (Table 4) identified 15 predicted binding residues across all compounds. PHE1238 formed interactions with every pose, while LEU1234 and ASN1241 were involved in all except that of Compound **1**. The PLIP interaction profiles closely mirrored the differences and similarities observed in the docking poses, complementing the visual representations in Figure 2.

In vitro evaluation confirmed the biological relevance of dimethoxycurcumin, which demonstrated potent activity across multiple PDAC cell lines. At a concentration of 10 µM, it substantially reduced cell viability and completely inhibited colony formation, key indicators of anti-proliferative efficacy and the ability to impair long-term clonogenic potential. The compound’s IC_50_ values ranged from 2.91 to 12.90 µM, depending on the cell line, highlighting consistent anti-tumor activity in a physiologically relevant concentration range. Although these IC_50_ values are higher in absolute terms (~1 order of magnitude) than that of the original query compound, BDBM50302828—reported to inhibit ABCC3 with an IC_50_ of 1.1 µM in a membrane vesicle-based assay—it is important to consider the distinct nature of the experimental models. BDBM50302828 was evaluated in a cell-free biochemical system designed to isolate transporter function [33], whereas dimethoxycurcumin’s activity was assessed using whole-cell viability assays. These assays inherently integrate additional layers of biological complexity, including compound uptake, intracellular metabolism, off-target interactions, and potential synergistic mechanisms. As such, a direct numerical comparison of IC_50_ values between these systems may be misleading; instead, the results should be interpreted qualitatively, with an emphasis on the translational relevance of whole-cell phenotypic responses.

Beyond its role as a transporter inhibitor, dimethoxycurcumin’s antioxidant properties may act synergistically to enhance its anticancer activity. Oxidative stress is a well-established driver of cancer progression and chemoresistance, with high levels of reactive oxygen species (ROS) influencing tumor cell survival, metastasis, and drug sensitivity [34]. While dimethoxycurcumin shows slightly lower antioxidant efficacy than curcumin, particularly in scavenging peroxyl radicals, it remains equally effective against superoxide radicals and exhibits similar activity in antioxidant pathways such as heme oxygenase-1 induction [35]. By simultaneously inhibiting ABCC3-mediated drug resistance and mitigating oxidative stress, dimethoxycurcumin may exert a dual mechanism of action, increasing its therapeutic potential.

Furthermore, the identification of dimethoxycurcumin extends the promising therapeutic potential of curcumin derivatives. Although the broad anticancer and antioxidant effects of curcumin have been extensively studied [36], specific targeting of ABCC3 has remained largely unexplored. Previous work has reported curcumin’s effects on ABCC1 (MRP1) [37] and ABCC5 (MRP5) [38], attributing transporter inhibition largely to its polyphenolic core structure. The present findings suggest ABCC3 as an additional, therapeutically relevant target for optimized curcumin analogs, highlighting the potential improvement of structural modifications, such as methylation, in enhancing transporter specificity and anticancer efficacy.

An acknowledged limitation in the clinical development of curcumin is its poor pharmacokinetic profile, including low solubility, limited bioavailability, and rapid systemic clearance [39]. Dimethoxycurcumin has been reported to exhibit improved metabolic stability [40], which may enhance its therapeutic performance compared to curcumin. However, further improvements in solubility and bioavailability may still be needed to optimize its clinical potential. Advanced delivery strategies such as cyclodextrin (CD) encapsulation, liposomes, and nanoparticle-based systems have shown promise in overcoming these limitations [41,42]. Cyclodextrins, cyclic oligosaccharides derived from starch degradation, form inclusion complexes with hydrophobic molecules, thereby improving their solubility and metabolic stability [43]. In particular, β-cyclodextrin and its derivatives, such as hydroxypropyl-β-cyclodextrin (HP-β-CD) and randomly methylated-β-cyclodextrin (RM-β-CD), have been shown to enhance the aqueous solubility and bioavailability of curcumin [44]. Applying similar formulation approaches to dimethoxycurcumin may further improve its pharmacokinetic properties and support its development as a clinically viable anticancer agent.

Moving forward, several promising avenues warrant further exploration. Firstly, elucidating the precise molecular interactions between dimethoxycurcumin and ABCC3 via experimental biochemical studies could provide invaluable insights into its mechanism of transporter inhibition. Secondly, assessing the compound’s specificity toward ABCC3 relative to other ABC transporters would better define its potential therapeutic window and minimize off-target effects. Finally, in vivo efficacy studies, particularly in relevant animal models of PDAC, combined with existing chemotherapeutics, could provide critical preclinical validation. Optimizing pharmacokinetics through strategic formulation approaches—such as those previously explored for curcumin analogs—could further enhance dimethoxycurcumin’s in vivo stability, improving its therapeutic potential.

## 5. Conclusions

Multidrug resistance (MDR) in pancreatic ductal adenocarcinoma (PDAC) remains a critical barrier to effective chemotherapy. A consensus fingerprint-based virtual screening approach was used to identify structurally diverse candidate inhibitors of the ABC transporter ABCC3. Following the initial hit selection, predicted pharmacokinetic properties were evaluated, and molecular docking was performed to explore potential binding modes. Docking results indicated that the compounds occupied a similar region within the ABCC3 translocation channel, though their binding poses varied in orientation and interaction patterns, reflecting scaffold diversity.

In vitro evaluation of the top-ranked compounds revealed variable levels of growth inhibition across pancreatic cancer cell lines. In contrast to the moderate or limited activity observed in most candidates, dimethoxycurcumin consistently demonstrated the strongest effects. In viability assays performed on BxPC-3 and HPAF-II cells, treatment with 10 μM dimethoxycurcumin led to a significant reduction in cell viability. In colony formation assays conducted using BxPC-3 cells, only dimethoxycurcumin resulted in complete suppression of colony growth. Based on this pronounced activity, a dose–response analysis was subsequently performed for dimethoxycurcumin across three cell lines—BxPC-3, HPAF-II, and CFPAC-1—confirming low micromolar IC_50_ values in all models.

The convergence of computational predictions with experimental outcomes supports the potential of dimethoxycurcumin to modulate ABCC3 activity and impair drug resistance mechanisms. In addition to transporter inhibition, the antioxidant properties associated with this compound may contribute to broader anti-tumor effects, including the modulation of oxidative stress and the enhancement of chemosensitivity.

These findings reinforce the value of integrated computational and experimental strategies in anticancer drug discovery and highlight dimethoxycurcumin as a promising candidate for further development. Future work should focus on validating ABCC3-specific inhibition, characterizing selectivity across ABC transporters, and assessing pharmacokinetic behavior in vivo, with attention to formulation techniques that may enhance bioavailability.

## Figures and Tables

**Table 1:** Top-ranked compounds from the Eurofins-VillaPharma library identified using ConFiLiS, based on consensus fingerprint similarity. The table includes Euclidean distances (Dist) and their corresponding z-score normalized values (Norm) for each fingerprint type: Avalon, Circular (ECFP6), and PubChem. The final consensus score was computed as the average of the three normalized distances. Only compounds with a consensus score below −2.5 were selected for further biological evaluation and are shown here.

CompoundID	Avalon Dist	Avalon Norm	Circular Dist	Circular Norm	PubChem Dist	PubChem Norm	Consensus Avg
**1**	9.747	−3.345	9.899	−2.963	9.747	−2.733	−3.013
**2**	10.149	−3.005	9.950	−2.854	10.488	−2.002	−2.620
**3**	12.369	−1.128	9.327	−4.196	10.050	−2.434	−2.586
**4**	12.961	−0.628	9.592	−3.626	9.055	−3.415	−2.557
**5**	12.806	−0.759	9.849	−3.072	8.775	−3.692	−2.508

**Table 2:** Structural similarity to known ABCC3 inhibitors from BindingDB. Each row shows the predicted compound, its most structurally similar known compound, and the corresponding Tanimoto similarity score using the Morgan (ECFP4) fingerprint. The synthesis of compounds **4** and **5** yielded racemic mixtures, as indicated by the wavy bond, used to represent stereochemical ambiguity.

Predicted Compound	Closest BindingDB Compound	ECFP4 Tanimoto Similarity
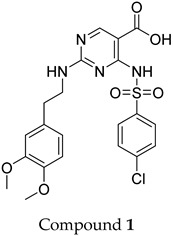	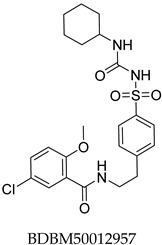	0.3810
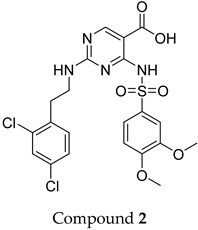	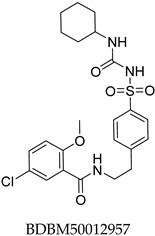	0.3222
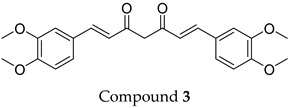	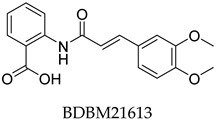	0.5556
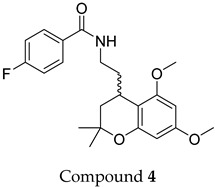	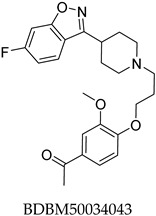	0.2637
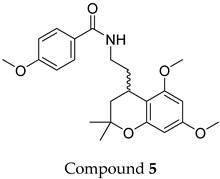	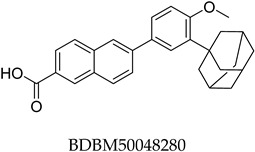	0.2703

**Table 3:** Predicted physicochemical and pharmacokinetic properties of selected ABCC3 inhibitor candidates using Schrödinger’s QikProp. For Compounds **4** and **5**, properties for both the R and S configurations were predicted. Properties include molecular weight (mol_MW), dipole moment, solvent-accessible surface areas (SASA for total surface area, FOSA for hydrophobic component, and FISA for hydrophilic component), polar surface area (PSA), number of rotatable bonds (#rotor), hydrogen bond donors and acceptors (donorHB, accptHB), lipophilicity (QPlogPo/w), aqueous solubility (QPlogS), Caco-2 cell permeability (QPPCaco), brain–blood barrier partitioning (QPlogBB), predicted human oral absorption (%Human Oral Absorption), and the number of violations of Lipinski’s Rule of Five and Jorgensen’s Rule of Three.

Properties	Compound 1	Compound 2	Compound 3	Compound 4 (R)	Compound 4 (S)	Compound 5 (R)	Compound 5 (S)
mol_MW	492.933	527.378	396.439	387.45	387.45	399.486	399.486
dipole	3.204	7.76	1.557	6.978	5.167	8.733	4.167
SASA	710.817	675.128	759.736	707.6	709.866	734.525	724.248
FOSA	235.466	169.765	438.151	390.289	393.435	483.11	479.647
FISA	210.469	218.471	82.343	46.848	46.83	46.498	38.271
PSA	145.07	139.776	81.826	58.054	58.196	66.341	66.298
#rotor ^1^	10	10	12	6	6	7	7
donorHB	2	2	0	1	1	1	1
accptHB	9.5	9.5	7	4.75	4.75	5.5	5.5
QPlogPo/w	3.164	3.262	4.215	5.281	5.285	5.128	5.157
QPlogS	−4.487	−4.104	−4.995	−6.636	−6.677	−6.464	−6.28
QPlogBB	−1.928	−1.698	−1.094	−0.177	−0.179	−0.356	−0.259
QPPCaco	25	21	1640	3561	3562	3588	4295
%Human Oral Absorption	71	57	100	100	100	100	100
RuleOfFive Violations	0	1	0	1	1	1	1
RuleOfThree Violations	0	1	0	1	1	1	1

^1^ #: number of rotatable bonds (rotors).

**Table 4:** Predicted protein–ligand interactions of selected ABCC3 hit compounds based on top blind docking poses. Docking scores (in kcal/mol), shown at the top of the table, reflect predicted binding affinities, with more negative values indicating stronger interactions. Each row summarizes interactions between a compound and specific ABCC3 residues, using the top-ranked pose from AutoDock Vina blind docking in the main translocation channel. Residue contacts were identified using PLIP, which detects key non-covalent interactions. A “Y” indicates at least one interaction with a residue; “N” indicates none. Stereochemical configurations (R/S) are specified for Compounds **4** and **5**.

	Compound 1	Compound 2	Compound 3	Compound 4 (R)	Compound 4 (S)	Compound 5 (R)	Compound 5 (S)
Vina Score	−9.54	−9.44	−8.07	−8.32	−8.29	−8.00	−8.21
ASN328	Y	Y	Y	Y	N	N	N
GLN363	Y	N	N	Y	N	N	N
SER364	N	Y	N	N	N	N	N
LEU367	N	Y	N	Y	Y	Y	N
ASN576	N	N	N	N	N	Y	N
ILE577	Y	Y	N	N	Y	Y	Y
ARG579	Y	N	N	N	N	N	N
LEU580	N	N	N	N	N	Y	N
ASN1204	Y	N	N	N	N	N	N
LEU1234	N	Y	Y	Y	Y	Y	Y
GLN1235	N	N	N	N	Y	N	Y
THR1237	N	N	Y	Y	N	N	N
PHE1238	Y	Y	Y	Y	Y	Y	Y
ASN1241	N	Y	Y	Y	Y	Y	Y
TRP1242	N	N	Y	N	N	Y	Y

## Data Availability

All data supporting the findings of this study are available from the corresponding author upon reasonable request. However, data derived from or related to the Eurofins-VillaPharma compound library are proprietary and cannot be shared due to confidentiality agreements.

## Abbreviations

The following abbreviations are used in this manuscript:

ABCC3	ATP-binding cassette subfamily C member 3
(HP/RM-β-)CD	(Hydroxypropyl/randomly methylated-β)-Cyclodextrin
CDK	Chemistry Development kit
ConFiLiS	Consensus Fingerprints for Ligand-based Screening
DAPI	4′,6-diamidino-2-phenylindole
Dist	Euclidean distance
ECFP(4/6)	Extended Connectivity Fingerprint (with a diameter of 4/6)
HCS	High-content screening
MDR	Multidrug resistance
MRP3	Multidrug resistance-associated protein 3
Norm	Normalized Euclidean distance
OD	Optical density
PDAC	Pancreatic ductal adenocarcinoma
PLIP	Protein–Ligand Interaction Profiler
ROS	Reactive oxygen species
